# Neurocognitive mechanisms of mathematics vocabulary processing in L1 and L2 in South African first graders: a functional near-infrared spectroscopy study

**DOI:** 10.1117/1.NPh.13.S1.S13005

**Published:** 2026-01-25

**Authors:** Hanrie S. Bezuidenhout, Parvin Nemati, Hadi Borj Khani, Candida Barreto, Elizabeth Henning, Mojtaba Soltanlou

**Affiliations:** a University of Johannesburg NRF SARChI Chair for Integrated Studies of Learning Language, Science and Mathematics in the Primary School, Johannesburg, South Africa; b Berlin University of Applied Sciences (HTW Berlin), Department of Energy and Information Engineering, Berlin, Germany; c Drexel University, School of Biomedical Engineering, Science and Health Systems, Philadelphia, Pennsylvania, United States; d University College London, UCL Institute of Education, Department of Psychology and Human Development, London, United Kingdom

**Keywords:** mathematics vocabulary, second language, language for mathematics, educational neuroscience, fNIRS, South Africa

## Abstract

**Significance:**

To learn mathematics, young children require accurate interpretations of mathematics vocabulary. When school language differs from children’s home language, mathematics performance often decreases. Little is known about cortical activation during mathematics vocabulary processing in different languages. Although behavioral data highlight a difference in L1 and L2 mathematics learning, neuroimaging insights will help us to better understand how and why there is a difference in children’s mathematical learning in multilingual societies.

**Aim and Approach:**

We investigated behavioral and brain responses (fNIRS) of 42 isiZulu and Sesotho (L1) first graders (6.75 to 7.83 years, 22 girls) who learn mathematics in English (L2) at school when they encounter mathematics vocabulary in L2 compared with L1 and mathematics vocabulary compared with object recognition in L1.

**Results:**

The results show that higher accuracy in the L1 mathematics vocabulary, as compared with the L2 mathematics vocabulary, comes with the costs of higher cognitive demands in the right superior and middle frontal gyri for first graders. Mathematics vocabulary required longer response time than object recognition and a higher activation in the right superior frontal gyrus. No parietal difference was observed between conditions.

**Conclusions:**

Neuroimaging revealed that children engaged additional frontoparietal regions when processing L1 mathematics vocabulary—patterns not detectable through behavioral measures alone. Increased frontal activation suggests that the interpretation of mathematics vocabulary in L2 is not yet automatized. This study demonstrates how educational neuroimaging refines interpretations of behavioral outcomes within multilingual contexts.

## 1 Introduction

Mathematics learning requires, among other skills, an accurate interpretation of mathematics vocabulary—key words, phrases, and abbreviations that are often used in mathematics instruction, assessments, and discussions.^1^^–^^3^ Interpreting mathematics vocabulary is more challenging for children who learn mathematics in a different language (L2) than their home language (Ll),^4^ which is common in multilingual societies such as South Africa. Unfamiliar technical terms (e.g., decrease), terms with more than one meaning (e.g., difference) and homonyms (e.g., sum), are examples that can make interpretation, understanding, and reasoning more difficult in L2.^5^ For instance, Bezuidenhout^6^ found that South African first graders, who receive mathematics instruction in L2, achieved higher scores on mathematics and mathematics vocabulary assessments in L1 (isiZulu/Sesotho—two of the official and most frequently used languages of South Africa) than in L2 (English), with concurrent and predictive associations between mathematics vocabulary and mathematics performance. She concluded that the children in that sample had more exposure to L1 mathematics vocabulary in their first 6 years and that the limited understanding of L2, their school language, directly influenced their lower performance in L2. According to the 2011 census,^7^ ∼77% of South Africans learn in a language different from their home language.^8^ Moreover, 40% of the world’s children learn in a second language,^9^ highlighting the need to understand differences between L1 and L2 learning.

To understand these differences, the context of general language structures, which is inherently needed for mathematical thinking,^1^^,^^10^ is vital. Mathematics tasks are often couched in word problems and require an understanding of both general language and vocabulary specific to mathematics.^5^ Language allows children to represent and process concepts^11^ by “looking” at concepts through a linguistic “lens.”^12^ Moreover, it is a tool^13^ that children can use to enlarge their existing representations of the world and to reason logically. Young children, who are more often exposed to everyday mathematical discussions with caregivers, parents, and teachers, are more likely to understand mathematics concepts than those with limited mathematical linguistic input.^14^

In this study, we focus on one specific component of language that has repeatedly been shown to contribute to the development of mathematics concepts,^1^^,^^2^ namely, mathematics vocabulary. Children with an elaborate mathematical vocabulary engage in discussions about comparison, descriptions of relationships, or properties of numbers or shapes, and describe mathematical procedures, which is why frequent exposure to mathematics vocabulary is vital.^14^ Children without mathematics vocabulary comprehension are unable to follow mathematics instruction or explain their reasoning for mathematics vocabulary.

Because of its direct impact on mathematics learning, mathematics vocabulary has been investigated in various age groups in different cultures.^2^ These studies not only emphasized the predictive validity of mathematics vocabulary^3^^,^^6^ and reciprocal relations between mathematics vocabulary and mathematics performance^1^ but also showed that mathematics vocabulary mediates the relationship between mathematics and general language.^10^ These mediating studies were mainly conducted in the Global North and mostly focused on one language. We situate our research within this mediator theory,^10^ which implies that mathematics vocabulary is an essential route through which general language ability influences mathematical understanding.

In multilingual classes, children navigate a variety of linguistic differences such as phonemes, morphology, sentence structures, clause complexity, word order, and length^15^ that can change the difficulty of mathematical texts and instruction in different languages.^10^ For instance, isiZulu and Sesotho are both transparent and compositional beyond the number 10 (for instance, “ten and one” for eleven), which is different to English. Thus, even though young L2 children may conceptually comprehend mathematics concepts and be able to demonstrate it in L1, their limited understanding of L2 may limit their mathematics performance when assessed in L2. To reduce linguistic load in multilingual classes, code-switching and translanguaging are used as pedagogical approaches. During code-switching,^16^ children preserve linguistic features of languages while switching between two defined linguistic structures within a sentence or by repeating sentences or words in a second language. Translanguaging^17^ dismantles distinct language features of named languages by spontaneously integrating spoken language into a single system. These methods allow teachers to simplify unknown syntax structures and to clarify mathematics vocabulary.

Little is known about how children process L2 mathematics vocabulary compared with L1. Draper et al.^18^ highlighted the underrepresentation of studies in majority countries such as sub-Saharan Africa, which is not only understudied but also home to many of the world’s children who learn in a second language. South Africa has 12 official languages, but most children learn mathematics in L1 until the third grade and then transition to English as the language of instruction in the fourth grade. Taylor and von Fintel^8^ argue that if children transition to L2 to learn mathematics—although education in L1 is suggested—it must not be without a solid understanding of concepts in L1, which is why a well-supported later transition is recommended. Yet, like many others, at the school where our research was conducted, children already receive instruction in English from the first grade, with some code switching and translanguaging.

To understand the phenomenon of learning mathematics in a second language, behavioral findings have been used to empirically identify limited mathematics vocabulary and for developing intervention programs^2^^,^^19^ by reporting accuracy, error rates, and reaction times. In addition, neuroimaging data can describe specific neural mechanisms such as differential cognitive load, resource allocation, and network engagement. As children grow and learn, neuroimaging provides a way to map how neural activation patterns change in location and intensity. Behavioral findings have shown that children’s mathematics performance in L1 is usually better than in L2;^6^ however, the underlying neural mechanisms are not clear. For instance, it might be due to more automatized processes that are related to the semantic long-term memory and brain regions on the left angular gyrus and temporoparietal cortex, or it might be due to more compact procedural processes that are related to fast step-by-step processes and the prefrontal cortex. Unfortunately, limited neuroimaging studies have compared differences in brain activation between L1 and L2 mathematics processing. In an fMRI study in young adults that investigated differences in L1 and L2 processing of mathematics tasks (i.e., numerical concept processing and exact calculation of multiplication and addition), Wang et al.^20^ found that compared with L1, mathematics tasks in L2 (English) involved higher neural activation in the left hemisphere, including the inferior frontal gyrus and Broca’s area, which is responsible for language processing. It is not yet clear if the same activation differences occur in children.

Other mathematics processing neuroimaging studies^21^ focused on number words or Arabic numerals. Yet, in South Africa, a comparison of L1 and L2 number words would be fruitless. A unique characteristic of South Africa—and most of the other previously colonized sub-Saharan countries—is that African speakers prefer to employ translanguaging by substituting long and difficult African number words with English number words when speaking in their home language. Because number words are also taught in English in most African classes, children rarely know the African number words. For this reason, we chose to investigate the processing of two logical qualifiers (i.e., more and less) in our study, rather than the processing of number words in L1 and L2. Note that the terms “logical qualifiers” and “numerical vocabulary” are interchangeably used in research. Previous neuroimaging studies that investigated logical quantifier processing were conducted in adults and only in one language. For instance, McMillan et al.^22^ found higher right IPS and dorsolateral prefrontal activation for comparative qualifiers (more and less) than for absolute quantifiers (all, some, and none). To the best of our knowledge, research in children’s understanding of these quantifiers is exclusively behavioral.^23^ Therefore, there is an unmet need for more neuroimaging studies to elucidate the brain mechanisms underlying that process in children.

Typically, mathematics is processed in the frontoparietal network.^24^^,^^25^ Different parts of the parietal regions, including the bilateral intraparietal sulcus (IPS) for quantity representation, the left angular gyrus (AG) for language-mediated processes during mathematics tasks (e.g., verbal short-term memory^25^) and the superior parietal lobule for spatially mediated processes, are involved in mathematical processing. Parietal activation for mathematical processing has been observed in different languages in different cultures.^24^ In addition, the prefrontal cortex, related to executive functions, serves a supportive domain-general role.^25^

In the context of Sweller’s cognitive load theory,^26^ we would expect that for young learners, assessments of L2 mathematics vocabulary would be a source of extraneous cognitive load in which participants overuse their prefrontal cognitive resources on decoding unfamiliar vocabulary and translating words. In comparison, in L1 assessments, the language barrier would be removed; the instructions would be instantly comprehensible and the extraneous cognitive load would drastically be reduced. Wang^20^ is an example of this interpretation in an adult sample. However, in a functional near infrared spectroscopy (fNIRS) study, Sugiura et al.^27^ found higher cortical activation in the bilateral superior/middle temporal and inferior parietal areas in 6- to 10-year-olds when processing general auditory L1 vocabulary than L2 vocabulary. They suggest that greater neural activation in L1 than L2 means that young children more easily process the phonology of L1, whereas phonologically unfamiliar L2 words are likely processed such as nonword auditory stimuli. Even though those studies provided evidence for the brain mechanisms underlying mathematical processing, it is still unclear how young children process mathematics vocabulary in their new school language (L2) compared with their more familiar home language (L1).

## 2 Current Study

This study aims to answer the following research questions: What are the possible differences in brain responses of first graders in multilingual settings when they encounter (1) mathematics vocabulary in L2 compared with L1; and (2) mathematics vocabulary compared with object recognition in L1? Our first hypothesis is built on the findings of Bezuidenhout^6^ that South African first graders achieve lower mathematics vocabulary scores in L2 than in L1. Based on their lower performance, we also expected higher cognitive engagement of the supporting prefrontal brain areas^20^ for L2 processing, as compared with L1. Because parietal areas are involved in mathematics processing in different languages^24^ and both L1 and L2 tasks tap into number processing, we did not expect parietal activation differences in this comparison. Second, we expected slower and less accurate responses during mathematics vocabulary than object recognition in L1. Because mathematics processing involves abstract symbolic processing and integration, whereas recognition of objects is largely automatic,^21^^,^^25^ we expected higher brain activation in the frontoparietal network for mathematics processing.

To answer our research questions, we monitored children’s bilateral frontoparietal activation differences of L1 (Sesotho/isiZulu) and L2 (English) during mathematics vocabulary tasks in first graders in South Africa.^28^ We aimed to understand (1) the brain responses in L1 and L2 processing of mathematics vocabulary and (2) brain activation during L1 processing of mathematics vocabulary and nonmathematics vocabulary (i.e., object recognition). For mathematics vocabulary, children compared two sets of objects to determine where it was more or less and used keypress to indicate their answer. Prerecorded auditory instructions were given, e.g., “where is more/less.” For object recognition, children recognized specific animals, e.g., “where is the bird?” and also used keypress to answer. We used fNIRS, which is a noninvasive brain imaging technique that allows studying brain activity in naturalistic settings.^29^ In addition, fNIRS is safe, cost-effective, and suitable for use with diverse populations, including young children.^30^

## 3 Materials and Methods

### 3.1 Participants

In a similar, though behavioral study, Bezuidenhout^6^ reported a large Cohen’s d effect size of 0.92. Based on Bezuidenhout,^6^ we calculated the sample size by using G*Power for paired t-tests (two tailed, alpha = 0.05, power = 0.9), which suggested a total of 12 children. However, to account for smaller effect sizes in fNIRS studies, we used a medium Cohen’s d effect size of 0.5, which suggested a total of 34 children for the current within-subject design.

Altogether, 42 children participated in the current study. Children would have been excluded from the study if they had learning difficulties, a diagnosed neurological or mental illness, diagnosed diabetes or blood pressure (hypertension), uncorrected diagnosed visual or auditory impairment, or had a serious head injury, such as a concussion, or if they did not finish the study. There were no such children. One of the challenges of an fNIRS study in sub-Saharan Africa is that some participants may have thick, dark hair or wear braids. Therefore, we asked them not to braid their hair when participating in the study.

The study was approved by the Research Committee of the University of Johannesburg which oversees research projects being conducted at the school, as well as the Ethics Committee of the Education Faculty of the University of Johannesburg (Sem 1-2022-035). The study was conducted according to the ethical guidelines and principles of the International Declaration of Helsinki. Children received incentives such as toys and stickers for their participation.

The study was conducted at the Cognition Lab at the University of Johannesburg, which is located next to the children’s school. The school was purposefully chosen to investigate the effect of learning mathematics in L2 in young children who have not been exposed to English as frequently as older children. When children’s school language differs from their home language, their mathematics performance often decreases.^6^ The isiZulu- and Sesotho-speaking first graders learn mathematics in English (L2) at this school. With enrolment in this teaching school, caregivers consent to their children’s participation in research experiments. However, this is one of the first neuroimaging studies that were conducted, and therefore, we also obtained written informed consent from the caregivers and participating children. The school is a quintile 2 school, which means that the school has been ranked as being in the second-poorest 20% of schools in the country based on the economic level of the surrounding community. These quintile poverty rankings, with quintile 1 being the lowest, are determined nationally according to the income, literacy, and unemployment levels of the community around the school, as well as certain infrastructural factors such as permanent versus temporary classes, classroom equipment, access to water and sanitation and safety measures.^31^

### 3.2 Procedure

In this within-subject study, we measured brain activation as well as six behavioral tasks to assess children’s general cognitive skills, of which two assessments were completed in both L1 and L2 for comparison purposes. Children whose caregivers consented to participation were collected from the school (which is on the premises of the university close to the lab) in groups of three to four children at a time and returned to their classes after participation. The experimenter introduced the children to the lab and explained the study. Although one child engaged in the behavioral assessments in a separate room, another completed the neuroimaging tasks. The remaining children were given toys in an adjacent room with a caregiver while waiting for their turn to participate. Completing all behavioral and neuroimaging tasks took approximately an hour per participant.

### 3.3 Behavioral Assessments

#### 3.3.1 Mathematics language

The Mathematics Vocabulary Test (MVT^19^) is a 24-item test for preschool and early grades children. It assesses numerical language qualifiers (more, many, just as many, fewer, few), comparative language (same size, bigger, tallest, biggest, big/large, tall, shortest, small, smaller, short) and spatial language (in between, first, last, on top of, behind, above, under, after, in front of). Each item requires the child to point to the picture that describes a specific concept. For instance: “Put your finger on the picture with more bugs.” None of the items requires exact quantitative knowledge, but only approximate numerical knowledge. One point is awarded for each correct response, and scores are calculated based on the total number of correct responses out of 24. Mathematics vocabulary was assessed in L1 (Sesotho/ isiZulu) and L2 (English) to compare the children’s performance in their home language and language of instruction.

#### 3.3.2 Mathematics

The Preschool Early Numeracy Skills Test^32^ is a 25-item numeracy test that assesses numeracy skills of children between 3 and 6 years. The items assess set comparison, numeral comparison, one-to-one correspondence, number order, identifying numerals, ordinality, and number combinations. Some items are multiple choice, and others are free response. Except for the first question, where a score out of five is determined, one point is awarded for each correct response, and scores are calculated based on the total number of correct responses out of 29. The test stops if the child answers three consecutive questions incorrectly. Mathematics performance was assessed in L1.

#### 3.3.3 Verbal short-term memory

The digit span task, as a subtest of the Wechsler Intelligence Scale for Children (WISC-IV), was used to assess how well children can recall spoken sequences of consonant digits (one digit per second). The test starts with sequences of two digits, which are increased by one if the child correctly recalls at least two out of three sequences. Each correct answer scored one point, and the total score is the sum of all points. Only forward recalls were included because of the children’s young age. Digit span was conducted in L1 and L2 to compare responses between the two languages.

#### 3.3.4 Nonverbal IQ

Subtest 3 of the Culture Fair Test^33^ is a 15-item pencil and paper subtest that assesses similarity recognition of 4- to 8-year-old children. Children identify as many similarities as they can, with a maximum of 15, within 90 seconds. For each item, one picture is presented with five similar pictures. The child sees a picture on the left side of a table and then selects one out of five pictures –the one that is exactly the same as the first picture. One point is awarded for each correct response, and scores are calculated based on the total number of correct responses out of 15. Instructions were given in L1.

#### 3.3.5 Listening comprehension

Gogo’s Dog (grandmother’s dog) with a listening comprehension scale^34^ based on the Shell–K listening comprehension protocol^35^ assesses listening comprehension. The test consists of one story (fiction), with 15 questions, ranging in difficulty from basic factual questions to questions, which require the child to infer answers from the text. The child responds orally, whereas the tester writes down their answers. This is not a timed test. One point is awarded for each correct response, and scores are calculated based on the total number of correct responses out of 15. Listening comprehension was assessed in L1.

### 3.4 fNIRS Tasks

During the neuroimaging tasks, the child wore an fNIRS cap, which was carefully fitted onto the head. Although calibration took place, the fNIRS cap was covered with an additional black cap to minimize light exposure. The experiment was conducted in a light-attenuated room, where all the lights were switched off before starting the calibration and experiment.

**Fig. 1:**
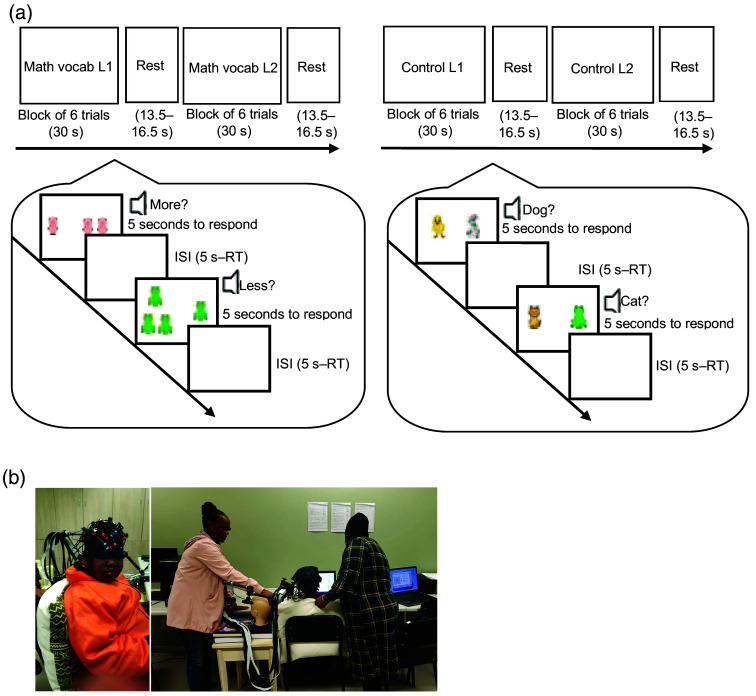
(a) Experimental tasks (i.e., mathematics vocabulary) and object recognition tasks (i.e., object recognition) were presented in a mixed block design in L1 and L2. Participants selected the correct picture corresponding to the auditory input with a key press. Note: ISI, inter-stimulus interval; RT, response time. (b) Preparing participants for the fNIRS tasks.^11^

The neuroimaging experiment consisted of two tasks: an experimental task containing 12 blocks of mathematics vocabulary processing, including six blocks in L1 and six blocks in L2; and a control object recognition task in L1, containing six blocks of object recognition (see Fig. 1). To balance our design, we also had a control condition in L2, but we did not include it in the analysis as it was not relevant to our research questions. Within the experimental tasks, the order of the L1 and L2 blocks was counterbalanced across children. Each experimental and object recognition task started with two practice blocks and each block consisted of 6 trials. After completing the practice blocks, the experimenter verbally checked with the children to make sure that they understood the instructions. The experimenters were three trained native isiZulu and Sesotho speakers, and verbal instructions were given in the children’s L1.

For each experimental trial, children saw two sets of pictures side-by-side on a computer screen, while hearing one of two words randomly: *more* or *less*. In half of the trials, they had to identify more items, whereas in the other half, they had to identify less items. By using keypress, children then chose the set of pictures related to the word they heard (see Fig. 1). They pressed key A for selecting the left set and key L for selecting the right set. For example, if one pig appeared on the left side of the screen and two pigs on the right, whereas the child heard “more”, the child had to press key L. Sets included one, two, three, or four animals, which were within the subitizing range.^36^ For each object recognition trial, children saw two different, but the same number of animals on the screen, heard the name of one of the animals on the screen, and selected the matching picture by using a keypress. In half of the trials, they had to select the left one, whereas in the other half, they had to select the right one. They needed to press key A for selecting the left one and key L for selecting the right one. Each trial was presented for a maximum of 5 s in both experimental and object recognition tasks. If the child responded faster than 5 s, a blank screen appeared for the remainder of the 5 s before the next trial appeared, as the inter-stimulus interval (ISI). Between each block, there was a resting time of 13.5 to 16.5 s with a jittering step of 0.5 seconds, which was randomly selected from a pool of seven possible lengths. Although feedback was given during the practice blocks, no feedback was given during the experimental questions. The neuroimaging experiment was conducted by using the program OpenSesame.^37^

## 4 fNIRS Recording, Preprocessing, and Analysis

A multichannel fNIRS device, the NIRScout system (NIRx Medical Technologies, LLC), was used to monitor and record cortical hemodynamic responses during the mathematics vocabulary and the object recognition tasks. There were 15 emitters that emit light at wavelengths between 760 and 850 nm, and 11 Avalanche Photodiode detectors. A sampling frequency of 4.1667 Hz and an average distance of 3 cm between optodes were used for data acquisition.

**Fig. 2:**
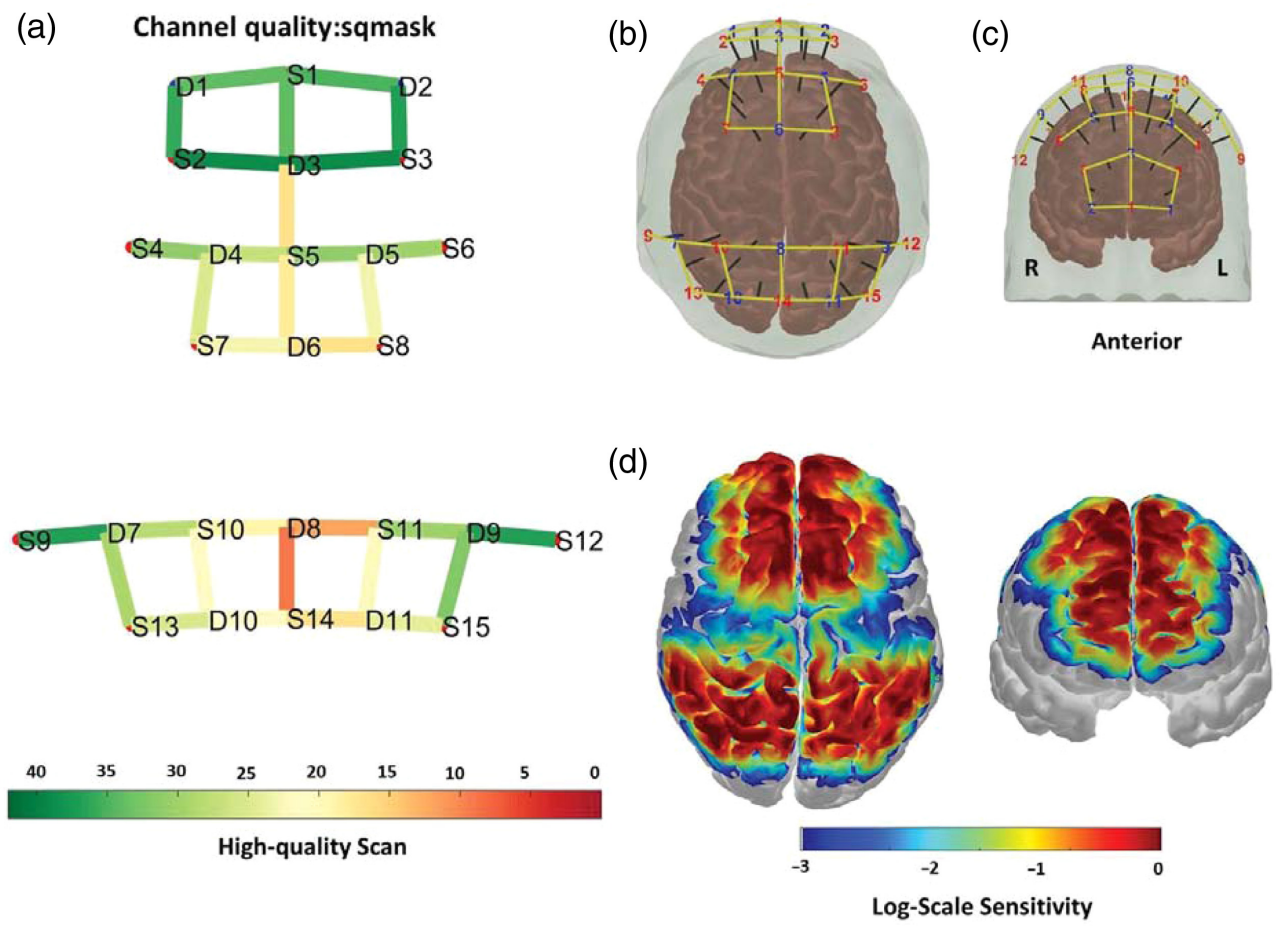
(a) Color bar indicates a signal quality map illustrating the number of participants per channel with a quality index exceeding 30%. (b) Superior view and (c) anterior view of the head model showing the positions of each source and detector, along with the projection of channels onto the cortical surface, highlighting the corresponding measurement locations and cortical regions. They show the positions of sources (red) and detectors (blue) distributed across the scalp. Yellow lines represent the channels formed by source–detector pairs, with black markers indicating the projections of these channels onto the cortical surface. (d) Sensitivity analysis based on the forward model simulation performed using AtlasViewer, illustrating the spatial sensitivity of the optodes to underlying cortical regions and the interaction of light propagation with brain tissue.^13^

At the beginning of the data processing, a rigorous quality assessment and control procedure was implemented. The QT-NIRS toolbox,^38^^,^^39^ which is available on the QT-NIRS GitHub (https://github.com/lpollonini/qt-nirs), was used to identify and exclude channels with inadequate data quality. Following Fonseca,^28^ channels below thresholds (SCI threshold = 0.5, Q threshold = 0.3, PSP threshold = 0.1) were excluded from subsequent analyses [Fig. 2(a)]. Across 42 participants (32 channels each; 1,344 total), 472 channels (35.1%) failed QC and were removed (mean 11.2 per participant), leaving 872 usable channels overall (64.9%), and no participants were excluded.

In Hernandez and Pollonini,^38^ these quality control steps were followed to retain channels that had acceptable quality metrics (QI > 30%, Cohen’s d > 0.5). We have estimated the MNI coordinates for the retained channel numbers and source–detector pairs using AtlasViewer^40^ in Table 1. Figures 2(b) and 2(c) illustrate the positions of each source and detector on the head model, presented from both superior and anterior views. The figure also includes the projection of each channel onto the cortical surface, providing a clear visualization of the measurement locations and their corresponding cortical regions. Figure 2(d) also shows the sensitivity analysis derived from the forward model simulation using AtlasViewer. This analysis quantifies the spatial sensitivity of the optodes to underlying cortical regions, providing a detailed visualization of how the light propagation interacts with the brain tissue and contributes to the measurement signals.

**Table 1:** Channel registration using AtlasViewer.

Ch	Src	Det	Ch Coord (Monte Carlo)	Ch Coord (MNI)	Area
1	1	1	144 149 214	−12 72 1	L medial orbital frontal
2	1	2	117 150 212	15 70 0	R medial orbital frontal
3	1	3	132 136 197	0 55 14	Superior medial frontal
4	2	1	151 144 202	−19 60 6	L superior frontal
5	2	3	143 128 200	−11 58 22	L superior frontal
6	3	2	108 140 200	24 58 10	R superior frontal
7	3	3	117 126 202	15 60 24	R superior frontal
8	5	3	130 108 199	2 57 42	Superior medial frontal
9	4	4	151 114 183	−19 41 36	L middle frontal
10	5	4	141 96 191	−9 49 54	L superior frontal
11	7	4	152 91 175	−20 33 59	L superior frontal
12	5	5	119 96 190	13 48 54	R superior frontal
13	6	5	107 113 180	25 38 37	R middle frontal
14	8	5	117 104 164	15 22 46	R superior frontal
15	5	6	131 84 175	1 33 66	Supplementary motor area
16	7	6	141 90 158	−9 16 60	L supplementary motor area
17	8	6	118 77 162	14 20 73	R supplementary motor area
18	9	7	191 103 102	−59 −40 47	L inferior parietal
19	10	7	164 92 103	−32 −39 58	L superior parietal
20	10	8	144 72 99	−12 −43 78	L postcentral
21	11	8	113 71 95	19 −47 79	R postcentral
22	11	9	88 82 97	44 −45 68	R superior parietal
23	12	9	73 105 98	59 −44 45	R inferior parietal
24	10	10	146 92 97	−14 −45 58	L superior parietal
25	11	11	99 78 86	33 −56 72	R superior parietal
26	13	7	160 111 96	−28 −46 39	L inferior parietal
27	13	10	155 110 86	−23 −56 40	L inferior parietal
28	14	8	129 87 97	3 −45 63	Precuneus
29	14	10	142 84 75	−10 −67 66	L precuneus
30	14	11	116 85 72	16 −70 65	R precuneus
31	15	9	88 102 89	44 −53 48	R inferior parietal
32	15	11	93 95 72	39 −70 55	R inferior parietal

R, right; L, left

To mitigate direct current shifts, baseline corrections were applied to the raw intensity signals (SD=3). After applying the Temporal Derivative Distribution Repair (TDDR) method to address motion artifacts and enhance data robustness,^41^ a bandpass filter of 0.01–0.2 Hz was applied.^42^ This filtering step was implemented to isolate and retain the frequency components corresponding to typical hemodynamic responses while removing low-frequency drifts and high-frequency noise. To further improve signal fidelity, minimize artifacts, and identify regressors, the AR-IRLS regression model^43^ was employed using the BrainAnalyzIR toolbox,^43^ as one of the most commonly used analysis tools.^44^

For the statistical analysis, a group-level approach was employed to investigate the effects of the conditions: mathematics vocabulary in L1 (L1Num), mathematics vocabulary in L2 (L2Num), and object recognition control condition in L1 (L1Con), as well as their contrasts, including L1Num–L2Num and L1Num–L1Con for each channel. A general linear mixed regression model was utilized to compute the beta coefficients for oxyhaemoglobin (HbO) and deoxyhaemoglobin (HbR) corresponding to each condition and contrast. To address the issue of multiple comparisons, the Benjamini-Hochberg False Discovery Rate (FDR) correction (pFDR) was applied to reduce false positivity. The analysis focused on identifying group-level effects across the conditions and their contrasts to provide insights into the distinct patterns of brain activation associated with each condition and contrast. The different brain regions were identified as activated based on their respective channels (source–detector pairs) with significant t-values (activation strength) and q-values (statistical significance). A significant increase/higher HbO and or a significant decrease/lower HbR are interpreted as an increase/higher brain activation in the corresponding channels.

## 5 Behavioral Analyses

In total, 42 children were included in the analyses. For the mathematics vocabulary tasks, 11 children were excluded from the accuracy analyses and 12 from the response time analyses in L1 and L2, respectively, due to technical problems with the fNIRS recording (see Table 2). Regarding demographic information, there were some missing data (5 for age and 20 for sex; see Table 2). Mean scores for all measures are reported in Table 2, and the differences between L1 and L2 for the mathematics language and digit span tasks were tested using paired t-tests.

**Table 2:** Descriptive statistics for study variables.

Measures	n	M	*SD*	Min. to Max.
Age	37	7.19	0.30	6.75 to 7.83
Sex (female)	22			
Nonverbal IQ	42	8.95	2.58	2 to 14
Listening comprehension	42	7.83	2.66	3 to 13
Mathematics	42	21.00	5.62	9 to 28
**Language 1**				
Mathematics language	42	21.10	3.05	18 to 30
Digit span forward	42	3.00	0.80	2 to 5
Accuracy in experimental condition	31	0.86	0.20	0.36 to 1.00
Accuracy in object recognition condition	31	0.86	0.13	0.50 to 0.95
RT in experimental condition	31	1836.20	355	1399 to 2620
RT in object recognition condition	31	1736	348	1208 to 2684
**Language 2**				
Mathematics language	42	21.20	3.54	15 to 29
Digit span forward	42	3.55	1.06	2 to 6
Accuracy in experimental condition	30	0.82	0.20	0.43 to 1.00
RT in experimental condition	30	1780	387	1175 to 2480

Bold p-values depict p<0.05

To test the behavioral performance for the first hypothesis (i.e., children respond slower and less accurately when mathematics vocabulary is presented in L2 than in L1), we conducted paired t-tests to compare response times and accuracy. Cohen’s d was used to report effect sizes and can be interpreted as d=0.2 indicating small, d=0.5 medium, and d=0.8 large effect.^45^^,^^46^ To test the behavioral performance for the second hypothesis (i.e., children respond slower and less accurately to mathematics vocabulary in L1 than the object recognition in L1), we conducted paired t-tests to compare response time and accuracy.

In addition, correlation analysis was used to test the relationships between study measures (i.e., mathematics vocabulary, mathematics language, mathematics, verbal short-term memory, nonverbal IQ, and listening comprehension) together with demographic factors and was reported for the interested readers (Appendix A). All the analyses were conducted by Jamovi (Version 2.5, 2024).

## 6 Results

### 6.1 Descriptive Statistics

Children demonstrated variability in performance across conditions, as reported in Table 2.

### 6.2 Mathematics Vocabulary in L1

**Fig. 3:**
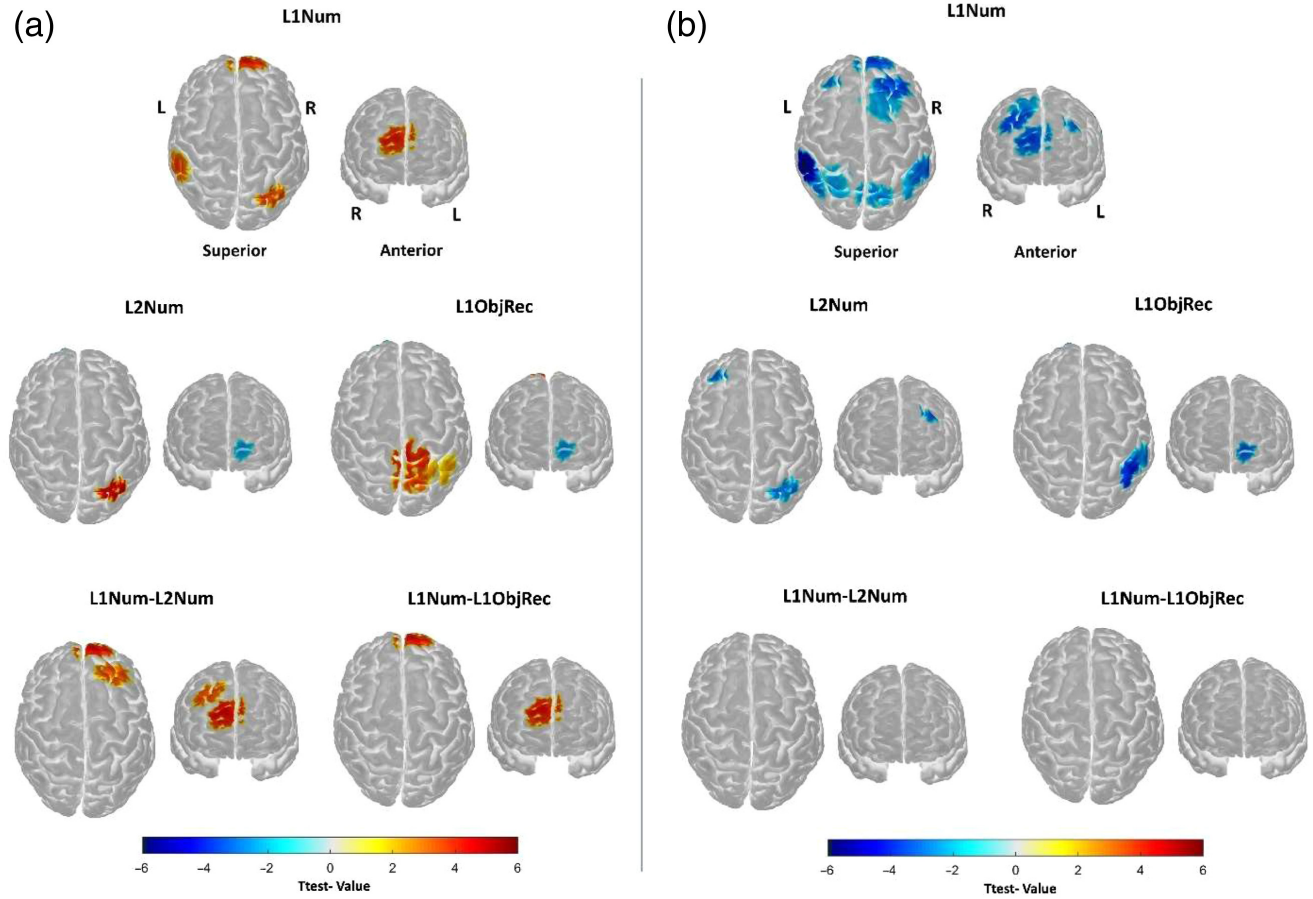
Spatial distribution of task-related brain activation for mathematical vocabulary in L1 (L1Num), mathematical vocabulary in L2 (L2Num), nonmathematical object recognition in L1 (L1Con), and their contrasts (L1Num-L2Num, L1Num-L1Con). (a) Regions with changes in HbO concentration. (b) Regions with changes in HbR concentration. Each brain map highlights significant cortical regions associated with the respective tasks and contrasts, overlaid on a standard brain template. Warm colors (yellow to red) represent positive t-values, whereas cool colors (blues) indicate negative t-values. The scale bar denotes t-values ranging from −6 to 6.^21^

The brain activation significantly increased in the right superior frontal gyrus, bilateral middle frontal gyri, bilateral inferior parietal gyri, the left superior parietal gyrus, and the right precuneus during mathematics vocabulary in L1 (Table 3 and Fig. 3).

**Table 3:** Significant channels in the three different conditions.

Region	Source	Detector	HbO/HbR	t	pFDR
**Mathematics vocabulary in L1**					
R superior frontal	3	3	HbO	4.23	0.001
R superior frontal	3	3	HbR	−3.74	0.004
L middle frontal	4	4	HbR	−3.77	0.004
R middle frontal	6	5	HbR	−4.15	0.002
R superior frontal	8	5	HbR	−2.99	0.029
L inferior parietal	9	7	HbO	3.78	0.004
L inferior parietal	9	7	HbR	−5.60	<0.001
L superior parietal	10	10	HbR	−2.80	0.048
R inferior parietal	12	9	HbR	−3.58	0.007
L inferior Parietal	13	7	HbR	−3.30	0.015
R precuneus	14	11	HbR	−3.06	0.026
R inferior parietal	15	9	HbR	−3.00	0.029
R inferior parietal	15	11	HbO	4.21	0.001
**Mathematics vocabulary in L2**					
L medial orbital frontal	1	1	HbO	−3.00	0.029
L middle frontal	4	4	HbR	−3.56	0.007
R inferior parietal	15	11	HbO	5.38	<0.001
R inferior parietal	15	11	HbR	−3.90	0.003
**Object recognition control in L1**					
L medial orbital frontal	1	1	HbO	−3.21	0.019
L medial orbital frontal	1	1	HbR	−3.80	0.004
R postcentral	11	8	HbO	4.55	0.001
R superior parietal	11	11	HbO	3.05	0.027
R inferior parietal	12	9	HbR	−3.18	0.020
R precuneus	14	11	HbO	4.05	0.002
R inferior parietal	15	9	HbO	2.85	0.044
R inferior parietal	15	9	HbR	−4.50	0.001
R inferior parietal	15	11	HbO	4.31	0.001

Each channel corresponds to a pair of a source and a detector. Source, the light source number; detector, the light detector number; HbO, oxyhaemoglobin; HbR, deoxyhemoglobin

### 6.3 Mathematics Vocabulary in L2

The brain activation significantly increased in the left medial orbital frontal and middle frontal gyri, and the right inferior parietal gyrus during mathematics vocabulary in L2 (Table 4 and Fig. 3).

**Table 4:** Significant channels in the two contrasts.

Region	Source	Detector	HbO/HbR	t	pFDR
**Mathematical vocabulary in L1 versus L2**					
R superior frontal	3	3	HbO	4.67	<0.001
R middle frontal	6	5	HbO	4.03	0.003
**Mathematical vocabulary in L1 versus object recognition control in L1**					
R superior frontal	3	3	HbO	4.41	0.001

Each channel corresponds to a pair of a source and a detector. Source, the light source number; detector, the light detector number; HbO, oxyhaemoglobin.

### 6.4 Object Recognition Control in L1

The brain activation significantly increased in the left medial orbital frontal gyrus, and the right postcentral, inferior, and superior parietal gyri, and precuneus during object recognition control in L1 (Table 3 and Fig. 3).

### 6.5 Mathematics Vocabulary in L1 versus L2

The results of the paired t-test revealed a significant difference in the accuracy of children performing mathematics vocabulary in L1 and L2, t(29)=1.71, p=0.049, Cohen’s d=0.31. Children had better performance in mathematics vocabulary in L1 (M=0.86, SD=0.20) than in L2 (M=0.82, SD=0.20). However, their response times during mathematics vocabulary in L1 and L2 showed no statistically significant difference, t(29)=1.04, p=0.153, Cohen’s d=0.19.

The contrast of mathematics vocabulary in L1 versus L2 showed higher brain activation in the right superior and middle frontal gyri (Table 4 and Fig. 3). The contrast on the right superior frontal gyrus was positively correlated with the Digit Span Forward in L1, r(31)=0.38, p=.034. This contrast reveals underlying brain activation of processing mathematical vocabulary in different languages. However, the beta value on the right superior frontal gyrus in L1 mathematics vocabulary condition was not correlated with Digit Span Forward in L1, r(31)=−0.01, p=.956.

### 6.6 Mathematics Vocabulary versus Object Recognition in L1

The results of the paired t-test revealed no significant difference in the accuracy of children performing mathematics vocabulary and object recognition in L1 t(30)=−0.15, p=0.558, Cohen’s d=−0.03. However, their response times during mathematics vocabulary and object recognition in L1 showed a statistically significant difference, t(30)=1.95, p=0.030, Cohen’s d=0.35. Children were slower during mathematics vocabulary in L1 (M=1848  ms, SD=355  ms) than object recognition in L1 (M=1736  ms, SD=348  ms).

The contrast of the mathematical vocabulary in L1 versus object recognition in L1 showed higher brain activation in the right superior frontal gyrus (Table 4 and Fig. 3). This contrast reveals underlying brain activation of mathematical processing.

### 6.7 Cognitive Measures

The results of the paired t-test comparing children’s performance in the mathematics language (MVT) in L1 (M=22.07, SD=3.05) and L2 (M=21.17, SD=3.54) showed no statistically significant difference, t(41)=1.63, p=0.112, Cohen’s d=0.25. However, their performance in verbal short-term memory (digit span) in L2 (M=3.55, SD=1.06) was statistically better than in L1 (M=3.00, SD=0.80), t(41)=−3.20, p=0.003, Cohen’s d=−0.49.

## 7 Discussion

The aim of this study was to investigate the neural correlates of mathematics vocabulary processing (i.e., more and less) in L1 (i.e., Sesotho/isiZulu) and L2 (i.e., English) by using fNIRS in first graders in South Africa. We hypothesized (i) slower and less accurate responses, as well as higher activation in the prefrontal cortex when mathematics vocabulary is processed in L2 than in L1; and (ii) slower and less accurate responses in mathematics vocabulary processing than object recognition, together with higher brain activation in the frontoparietal network in L1. In the first hypothesis, we did not expect a difference in parietal activation between L1 and L2 for mathematics vocabulary processing.

### 7.1 Mathematics Vocabulary in L1 and L2

The results of our study show that first graders who learn mathematics in L2 have higher activation in the right superior and middle frontal gyri for L1 mathematics vocabulary processing than for L2. Higher activation shows that higher accuracy in the L1 mathematics vocabulary task comes with the costs of higher executive function demands. Although children responded, as hypothesized, more accurately in the L1 fNIRS mathematics vocabulary task, their response time did not differ. Contrary to our hypothesis, children showed higher activation in the brain areas that support domain executive functions^25^ for the L1 mathematics vocabulary task. This counterintuitive neuroimaging finding challenges a simple application of cognitive load theory^26^ and points to more complex cognitive mechanisms, which require more nuanced interpretation than what behavioral findings alone may have suggested. This complexity has been shown in the field of mathematical cognition. For example, Soltanlou et al.^47^ showed that behavioral improvement after mathematical training in children with mathematical difficulties led to brain activation increase in several regions in the frontoparietal network, unlike the brain activation decrease after mathematical training in neurotypical children.^29^ Learning from those findings, in the current study, it seems that first graders are not yet automatized in processing mathematical vocabulary in L1. Higher prefrontal activation in L1 may not signify more effort, but better, more efficient, and deeper engagement in higher-order thinking.^48^ The higher prefrontal activation reflects that participants were expertly engaged with the task because they have richer L1 phonological and semantic associations with vocabulary in L1,^27^ which they can integrate with mathematics concepts stored in the long-term memory.^49^ This means that the participants in our study use L1 as the language of reasoning, abstract thought, and nuanced understanding, whereas English L2 is a weaker, more superficial linguistic code. Because young children are at the learning stage of those concepts and are not yet masters, they still heavily rely on domain-general cognitive resources for learning mathematical concepts, especially in L2. Although our behavioral results indicated that first graders performed better in L1 mathematics vocabulary tasks, neuroimaging helped us understand that the children engaged more deeply in their expert language, whereas L2 activation showed an overwhelmed brain with superficial processing.

Our behavioral results support the neuroimaging findings. The relationships between mathematics and mathematics language (Appendix A), together with the differences between L1 and L2 mathematics vocabulary performance, lead us to conclude that although children may understand mathematical concepts, they may not yet be able to integrate L2 vocabulary with mathematics conceptual networks. In this study, isiZulu- or Sesotho-speaking learners have not yet automatized or integrated English mathematics vocabulary into a coherent mathematical conceptual network as much as L1 vocabulary. This raises the question whether children begin to use L1 mathematics vocabulary as a mediator for the interpretation of L2 mathematics vocabulary, as suggested by Wang.^20^ Further research is needed to investigate whether there exists a developmental shift in how L1 mediates L2 meaning in childhood compared with adulthood.

Furthermore, children performed better on L2 digit span tasks (i.e., the recall of number sequences) than in L1, which was associated with other cognitive skills (i.e., listening comprehension, numeracy, and similarity recognition). These findings can be explained by the practice of translanguaging^17^ in South Africa, where most African speakers use English number words in their everyday discussions rather than number words in their home languages. This means that children used a reduced cognitive load^26^ for the more familiar English words compared with less familiar L1 number words.

### 7.2 Object Recognition and Mathematics Vocabulary in L1

According to our hypothesis, mathematics vocabulary required longer response times than object recognition and a higher cognitive demand for supportive functions in the right superior frontal gyrus. However, contradictory to our hypothesis, there was no difference in their accuracy during these two tasks. As we expected, mathematics vocabulary tasks had slower responses and higher frontal activation, suggesting their demand for higher domain-general cognitive processes. This means that the mathematics vocabulary task was more difficult for children than the object recognition tasks. Contrary to our expectations, there was no parietal difference observed in the contrast of mathematics vocabulary and object recognition tasks (Fig. 3). Although we observed right IPS activation during mathematics vocabulary in L1, object recognition in L1 led to a high activation in the right superior parietal lobule. The IPS, particularly in the right hemisphere, is involved in processing magnitudes during almost any kind of numerical cognition task,^50^ which is why mathematics vocabulary processing activates the right IPS. The right superior parietal lobe is related to visuospatial processing and supports spatial working memory (e.g., remembering object positions), which is why this area was activated in object recognition. These two activated regions subtracted from each other in the contrast analysis, resulting in no difference in the parietal region. One possible reason that the activation was subtracted might be the low spatial resolution of fNIRS.^51^ Note that more specific brain regions, such as inferior temporal lobe, are involved in object recognition; however, due to a limited number of optodes, we were not able to record that region in the current study.

### 7.3 Challenges and Limitations

As one of the first fNIRS studies conducted with children in South Africa, this project presented unique opportunities and challenges. A key step was training isiZulu- and Sesotho-speaking research assistants to administer the fNIRS tasks, which extended the data collection period but ultimately built valuable local research capacity. Although some data loss occurred due to the learning curve of new research assistants, the experience strengthened future protocols. Another challenge faced on this study was related to the limitation of fNIRS devices to handle traditional hair braiding, which can interfere with optode–scalp contact in parietal regions. This was addressed by inviting children to participate without braids, allowing for improved data quality. These experiences underscore the pressing need for more inclusive fNIRS devices that accommodate diverse hair types and cultural practices, ensuring equitable participation in neuroscience research worldwide.

Furthermore, as all participants were recruited from a single school and from one isiZulu and one Sesotho class, the study could not account for classroom-level effects using hierarchical modeling due to the limited number of clusters. Although this represents a methodological consideration, it is unlikely to have substantially impacted the primary findings. In our analysis of brain–behavior correlations, we focused on the relationship between frontal activation for L1 mathematics vocabulary and working memory. Although other domain-general cognitive functions were not assessed, this analysis provides a targeted insight into the cognitive mechanisms supporting mathematical processing. Finally, although some missing demographic information and the exclusion of a subset of children due to technical issues may have modestly reduced statistical power, the findings remain informative and offer a valuable foundation for future, larger-scale investigations.

## 8 Conclusion

As one of the first educational neuroscientific studies in sub-Saharan countries, the fNIRS findings provided valuable insights into the neurocognitive mechanisms underlying mathematics vocabulary interpretation in different languages. The higher frontal activation during mathematics vocabulary in L1 suggests that young children are not yet automatized in the interpretation of mathematics vocabulary in L2. In our study, neuroimaging data made it clear that children used additional resources for L1 processing, which we would not be able to identify with behavioral data alone. This type of neuroimaging result is used to inform educational practitioners to tailor their pedagogical tools and to develop targeted educational strategies in multilingual classes. Understanding neural correlates informs early detection of atypical development (e.g., mathematics learning difficulties) and the development of effective interventions.

Considered in the context of mathematics vocabulary as a mediator between language and mathematics,^10^ our educational neuroscientific findings highlight the importance of targeted vocabulary scaffolding in which mathematics vocabulary is explicitly taught in context. For instance, relational words (e.g., more than, less than) should be well established before introducing arithmetic operations and procedures. Newly learnt words must be repeated and rehearsed in various contexts by systematically replacing L1 words in translanguage sentences with L2 words. To reduce the cognitive load during teaching and assessments in L2, linguistic complexity can be simplified through translanguaging^17^ and code switching.^16^ Furthermore, teachers could sequence instruction so that the linguistic complexity does not overshadow conceptual content. To reduce the cognitive load when learning in more than one language, visual–verbal integration should be used by consistently pairing mathematics words with symbols, diagrams, and gestures to reinforce meaning.^26^ Our findings also highlight the neurocognitive tolls during L1 at the school entry, which further question the use of L2 for mathematics instruction from school entry.

Draper et al.^18^ call for a better understanding of cognitive development in countries with diverse cultural and social characteristics. Although South African children learn mathematics in L1, their primary language for mathematics is English. This is also the case in several other colonized sub-Saharan countries and the Global South, where their academic language has been contributed to by the colonizer’s language. Taken together, we conclude that neuroimaging studies in the majority of countries are vital to understand more than simply what children can do by observing their behavior but also to describe the process of how their brains are learning. Neuroimaging provides objective, mechanistic insights that behavioral studies alone cannot, especially in culturally unique environments such as South Africa.

## 9 Appendix A

The correlations show a relationship between mathematics and mathematics vocabulary. Mathematics exhibited significant positive correlation with mathematics language in L1 and L2. There was also a positive significant correlation between mathematics language in L1 and L2. Accuracy of mathematics vocabulary performance in L1 showed a significant positive correlation with mathematics vocabulary performance in L2 (Table 5).

**Table 5:** Correlations among measurements.

Measures	1	2	3	4	5	6	7	8	9	10	11	12	13	14
1. Age	—													
2. Nonverbal IQ	0.04	—												
3. Listening comprehension	0.21	0.42**	—											
4. Mathematics	−0.03	0.41**	0.67***	—										
**Language 1**														
5. Mathematics language	0.32	0.22	0.52***	0.32*	—									
6. Digit span forward	0.17	0.18	0.23	0.27	0.12	—								
7. Accuracy in experimental condition	0.07	0.45*	0.24	0.33	0.07	0.37*	—							
8. RT in experimental condition	0.18	0.17	0.37*	0.45*	0.30	0.02	0.06	—						
9. Accuracy in object recognition condition	0.14	0.001	0.13	0.21	0.02	0.24	0.56***	0.06	—					
10. RT in object recognition condition	−0.12	−0.13	0.07	0.20	0.22	0.02	−0.21	0.59***	−0.30	—				
**Language 2**														
11. Mathematics language	.26	.17	.41**	.35*	.41**	.06	.11	.13	.21	.02	—			
12. Digit span forward	0.22	0.31*	0.42**	0.37*	0.30	0.32*	0.19	0.19	0.41**	0.21	0.41**	—		
13. Accuracy in experimental condition	0.20	0.35	0.34	0.34	0.61	0.34	0.87***	0.05	0.61***	−0.21	0.31	0.16	—	
14. RT in experimental condition	−0.08	0.01	0.18	0.29	0.16	0.08	0.05	0.67***	0.07	0.68***	0.02	−0.08	0.08	—

* p<.05, ** p<.01, *** p<.001

Degrees of freedom slightly varied for correlations among variables based on our records (see Table 2).

## Data Availability

The data that support the findings of this study are available on request from the corresponding author (hsbezuidenhout@uj.ac.za).

## References

[1] Bleses D. et al., “General and math vocabulary contributions to early numeracy skills in a large population-representative sample,” Front. Dev. Psychol. 1, 1279691 (2023). 10.3389/fdpys.2023.1279691

[r2] Lin X. Peng P. Zeng J., “Understanding the relation between mathematics vocabulary and mathematics performance: a meta-analysis,” Elementary School J. 121(3), 504–540 (2021). 10.1086/712504

[3] Purpura D. J. Reid E. E., “Mathematics and language: individual and group differences in mathematical language skills in young children,” Early Childhood Res. Quart. 36, 259–268 (2016). 10.1016/j.ecresq.2015.12.020

[4] Tshikondela G. et al., “Grade 2 learners’ performance on an interview-based numeracy test: assessments in English and Tshivenda,” Afr. J. Res. Math. Sci. Technol. Educ. 29(2), 143–157 (2025). 10.1080/18117295.2024.2434802

[5] Powell S. R. et al., “The word-problem solving and explanations of students experiencing mathematics difficulty: a comparison based on dual-language status,” Learn. Disability Quart. 45(1), 6–18 (2020). 10.1177/0731948720922198

[6] Bezuidenhout H. S., “Diagnostic test for number concept development during early childhood,” South Afr. J. Childhood Educ. 8(1), 1–10 (2018). 10.4102/sajce.v8i1.584

[7] Statistics South Africa, Statistical Release: Census 2011, Statistics South Africa, Pretoria (2012).

[8] Taylor S. von Fintel M., “Estimating the impact of language of instruction in South African primary schools: a fixed effects approach,” Econ. Educ. Rev. 50, 75–89 (2016).0272-7757 10.1016/j.econedurev.2016.01.003

[9] Maurer B. et al., Languages Matter: Global Guidance on Multilingual Education, UNESCO (2025).

[10] Ho J. et al., “Relation between general vocabulary knowledge and early numeracy competence: the mediating role of mathematical language,” J. Exp. Child Psychol. 252, 106145 (2025). JECPAE 0022-0965 10.1016/j.jecp.2024.106145 39673823

[11] Vygotsky L. S., Thought and Language, Kozulin A., Ed., MIT Press, Cambridge (1986).

[12] Gentner D. Goldin-Meadow S., Eds., “Whither whorf,” in Language in Mind: Advances in the Study of Language and Thought, pp. 3–14, MIT Press (2003).

[13] Levine S. Baillargeon R., “Different factors of language in numerical development: exact number and individuation,” in Core Knowledge and Conceptual Change, Barner D. Baron S., Eds., pp. 127–150, University Press, Oxford (2016).

[14] Levine S. C. et al., “What counts in the development of young children’s number knowledge?” Dev. Psychol. 46(5), 1309 (2010). DEVPA9 0012-1649 10.1037/a0019671 20822240 PMC2998540

[15] Dowker A. Nuerk H. C., “Linguistic influences on mathematics,” Front. Psychol. 7, 1035 (2016).1664-1078 10.3389/fpsyg.2016.01035 27462286 PMC4940406

[16] Setati M. Adler J., “Between languages and discourses: language practices in primary multilingual mathematics classrooms in South Africa,” Educ. Stud. Math. 43(3), 243–269 (2001). EDSMAN 0013-1954 10.1023/A:1011996002062

[17] Otheguy R. García O. Reid W., “Clarifying translanguaging and deconstructing named languages: a perspective from linguistics,” Appl. Linguistics Rev. 6(3), 281–307 (2015). 10.1515/applirev-2015-0014

[18] Draper C. E. et al., “Publishing child development research from around the world: an unfair playing field resulting in most of the world’s child population underrepresented in research,” Infant Child Dev. 32(6), e2375 (2022). 10.1002/icd.2375

[19] Bezuidenhout H. S. et al., “Measuring mathematics vocabulary in a multilingual context: identifying children with limited mathematics vocabulary in South Africa,” Dev. Psychol. (2025). DEVPA9 0012-1649 10.1037/dev0001941

[20] Wang Y. et al., “Mathematical and linguistic processing differs between native and second languages: an fMRI study,” Brain Imaging Behav. 1(3), 68–82 (2007). 10.1007/s11682-007-9007-y

[21] Arsalidou M. Taylor M. J., “Is 2 + 2 = 4? Meta-analyses of brain areas needed for numbers and calculations,” NeuroImage 54(3), 2382–2393 (2011). NEIMEF 1053-8119 10.1016/j.neuroimage.2010.10.009 20946958

[22] McMillan C. T. et al., “Neural basis for generalized quantifier comprehension,” Neuropsychologia 43(12), 1729–1737 (2005). NUPSA6 0028-3932 10.1016/j.neuropsychologia.2005.02.012 16154448

[23] Carey S., The Origin of Concepts, Oxford University Press, Oxford (2009).

[24] Arsalidou M. et al., “Brain areas associated with numbers and calculations in children: Meta-analyses of fMRI studies,” Dev. Cognit. Neurosci. 30, 239–250 (2018). 10.1016/j.dcn.2017.08.002 28844728 PMC6969084

[25] Dehaene S. et al., “Arithmetic and the brain,” Curr. Opin. Neurobiol. 14(2), 218–224 (2004). COPUEN 0959-4388 10.1016/j.conb.2004.03.008 15082328

[26] Sweller J., “Cognitive load theory,” in Psychology of Learning and Motivation, Mestre J. Ross B. H., Eds., Vol. 55, pp. 37–76, Academic Press (2011).

[27] Sugiura L. et al., “Sound to language: different cortical processing for first and second languages in elementary school children as revealed by a large-scale study using fNIRS,” Cereb. Cortex 21(10), 2374–2393 (2011). 53OPAV 1047-3211 10.1093/cercor/bhr023 21350046 PMC3169662

[28] Fonseca K. et al., “Individual differences modulate the neural correlates of fraction processing in primary school children in South Africa,” Cognitive Development 76, 101636 (2025). 10.1016/j.cogdev.2025.101636

[29] Soltanlou M. et al., “Applications of functional near-infrared spectroscopy (fNIRS) in studying cognitive development: the case of mathematics and language,” Front. Psychol. 9, 277 (2018).1664-1078 10.3389/fpsyg.2018.00277 29666589 PMC5891614

[30] Pinti P. et al., “The present and future use of functional near‐infrared spectroscopy (fNIRS) for cognitive neuroscience,” Ann. N. Y. Acad. Sci. 1464(1), 5–29 (2020). ANYAA9 0077-8923 10.1111/nyas.13948 30085354 PMC6367070

[31] South African Department of Basic Education, “Understanding school quintiles,” (2023). https://www.education.gov.za/Resources/FAQs/SchoolQuintiles.aspx

[32] Purpura D. J. Lonigan C. J., “Early numeracy assessment: the development of the preschool early numeracy scales,” Early Educ. Dev. 26(2), 286–313 (2015). 10.1080/10409289.2015.991084 25709375 PMC4335720

[33] Cattell R. B. Cattell A. K. S., Culture Fair Test, Institute for Personality and Ability Testing, Champaign, IL (1959).

[34] Ragpot L. Brink S., Grade 1 Listening Comprehension Scale, University of Johannesburg Centre for Childhood Education Practice (CEPR), Johannesburg (2016).

[35] Snow C. E. Burns M. S. Griffin P., Preventing Reading Difficulties in Young Children, Department of Education, Washington, DC (1998).

[36] Starkey P. Cooper R. G. Jr., “The development of subitizing in young children,” Br. J. Dev. Psychol. 13(4), 399–420 (1995). 10.1111/j.2044-835X.1995.tb00688.x

[37] Mathôt S. Schreij D. Theeuwes J., “OpenSesame: an open-source, graphical experiment builder for the social sciences,” Behav. Res. Methods 44(2), 314–324 (2012). 10.3758/s13428-011-0168-7 22083660 PMC3356517

[38] Hernandez S. M. Pollonini L., “NIRSplot: a tool for quality assessment of fNIRS scans,” in Biophotonics Congress: Biomed. Opt. 2020 (Translational, Microscopy, OCT, OTS, BRAIN), p. BM2C.5 (2020). 10.1364/BRAIN.2020.BM2C.5

[39] Pollonini L. Bortfeld H. Oghalai J. S., “PHOEBE: a method for real time mapping of optodes-scalp coupling in functional near-infrared spectroscopy,” Biomed. Opt. Express 7(12), 5104–5119 (2016). BOEICL 2156-7085 10.1364/BOE.7.005104 28018728 PMC5175555

[40] Aasted C. M. et al., “Anatomical guidance for functional near-infrared spectroscopy: AtlasViewer tutorial,” Neurophotonics 2(2), 020801 (2015). 10.1117/1.NPh.2.2.020801 26157991 PMC4478785

[41] Fishburn F. A. et al., “Temporal derivative distribution repair (TDDR): a motion correction method for fNIRS,” NeuroImage 184, 171–179 (2019). NEIMEF 1053-8119 10.1016/j.neuroimage.2018.09.025 30217544 PMC6230489

[42] Scholkmann F. et al., “A review on continuous wave functional near-infrared spectroscopy and imaging instrumentation and methodology,” NeuroImage 85, 6–27 (2014). NEIMEF 1053-8119 10.1016/j.neuroimage.2013.05.004 23684868

[43] Santosa H. et al., “Quantitative comparison of correction techniques for removing systemic physiological signal in functional near-infrared spectroscopy studies,” Neurophotonics 7(3), 035009 (2020). 10.1117/1.NPh.7.3.035009 32995361 PMC7511246

[44] Yücel M. A. et al., “fNIRS reproducibility varies with data quality, analysis pipelines, and researcher experience,” Commun. Biol. 8(1), 1149 (2025). 10.1038/s42003-025-08412-1 40760004 PMC12322209

[45] Cohen J., Statistical Power Analysis for the Behavioral Sciences, 2nd ed., Lawrence Erlbaum, Hillsdale, NJ (1988).

[46] Cragg L. et al., “Direct and indirect influences of executive functions on mathematics achievement,” Cognition 162, 12–26 (2017). CGTNAU 0010-0277 10.1016/j.cognition.2017.01.014 28189034

[47] Soltanlou M. et al., “Training causes activation increase in temporo-parietal and parietal regions in children with mathematical disabilities,” Brain Struct. Funct. 227(5), 1757–1771 (2022). 10.1007/s00429-022-02470-5 35257218 PMC9098620

[48] Dehaene S., How We Learn: The New Science of Education and the Brain, Penguin UK (2020).

[49] Linck J. A. et al., “Working memory and second language comprehension and production: a meta-analysis,” Psychon. Bull. Rev. 21(4), 861–883 (2014). PBUREN 1069-9384 10.3758/s13423-013-0565-2 24366687

[50] Hyde D. C., “The emergence of a brain network for numerical thinking,” Child Dev. Perspect. 15(3), 168–175 (2021). 10.1111/cdep.12418

[51] Barreto C. Soltanlou M., “Functional near-infrared spectroscopy as a tool to assess brain activity in educational settings: an introduction for educational researchers,” South Afr. J. Childhood Educ. 13(1), 1138 (2022). 10.4102/sajce.v12i1.1138

